# Cell wall polysaccharides of Gram positive ovococcoid bacteria and their role as bacteriophage receptors

**DOI:** 10.1016/j.csbj.2021.07.011

**Published:** 2021-07-14

**Authors:** Katherine Lavelle, Douwe van Sinderen, Jennifer Mahony

**Affiliations:** School of Microbiology & APC Microbiome Ireland, University College Cork, Western Road, Cork T12 YT20, Ireland

**Keywords:** *Lactococcus*, *Streptococcus*, *Staphylococcus*, *Enterococcus*, Cell envelope, Rhamnose-glucose polymers, Phage-host interactions

## Abstract

Gram-positive bacterial cell walls are characterised by the presence of a thick peptidoglycan layer which provides protection from extracellular stresses, maintains cell integrity and determines cell morphology, while it also serves as a foundation to anchor a number of crucial polymeric structures. For ovococcal species, including streptococci, enterococci and lactococci, such structures are represented by rhamnose-containing cell wall polysaccharides, which at least in some instances appear to serve as a functional replacement for wall teichoic acids. The biochemical composition of several streptococcal, lactococcal and enterococcal rhamnose-containing cell wall polysaccharides have been elucidated, while associated functional genomic analyses have facilitated the proposition of models for individual biosynthetic pathways. Here, we review the genomic loci which encode the enzymatic machinery to produce rhamnose-containing, cell wall-associated polysaccharide (Rha cwps) structures of the afore-mentioned ovococcal bacteria with particular emphasis on gene content, biochemical structure and common biosynthetic steps. Furthermore, we discuss the role played by these saccharidic polymers as receptors for bacteriophages and the important role phages play in driving Rha cwps diversification and evolution.

## Graphical abstract

**Figure:**
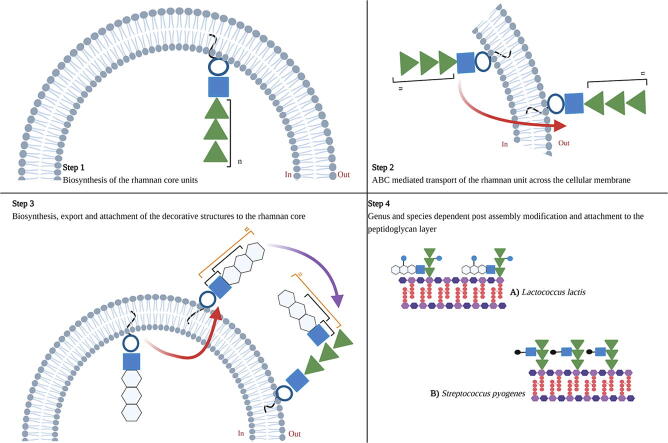

## 1 Introduction

**Fig. 1:**
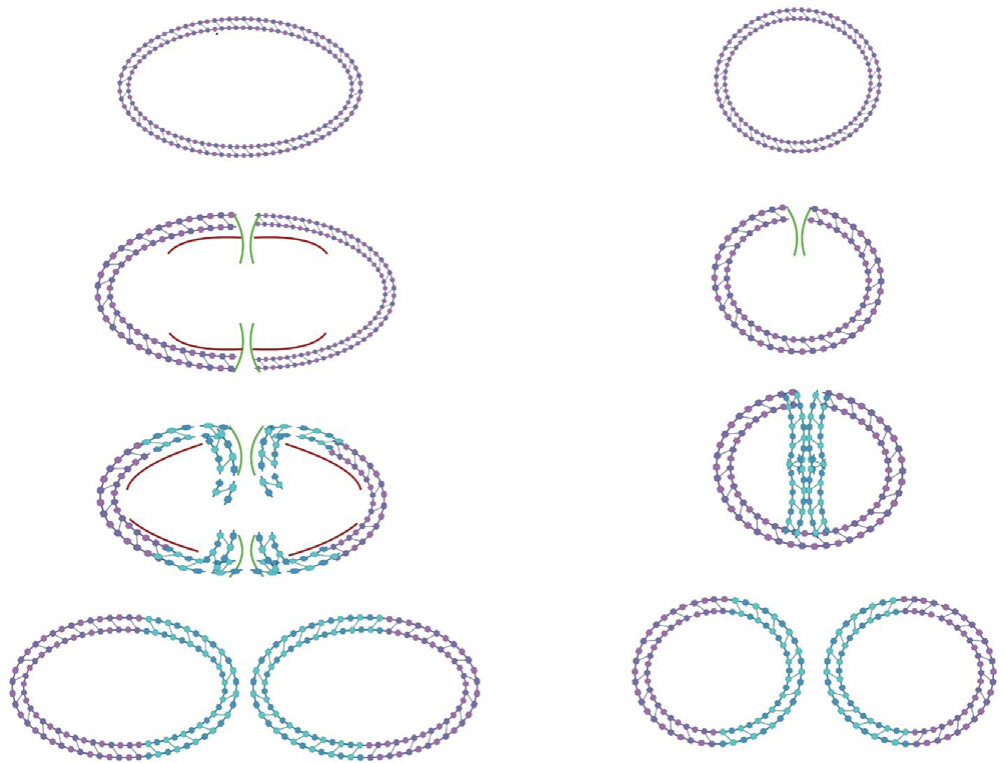
Outline overview of differential peptidoglyan synthesis in **(Left)** ovococcoid and **(Right)** coccoid species. Ovococcoid species employ a dual mode of nacent peptidoglycan synthesis – peripheral (red indicator) and septal (green idicator) which results in the formation of an equatorial ring, characterisitic of ovococcoid cells. In contrast, the so called “true” cocci employ a single, septal mode of division. Adapted from Zapun *et al*., [1]. Image created with BioRender.com. (For interpretation of the references to colour in this figure legend, the reader is referred to the web version of this article.)

The Gram-positive bacterial cell wall contains a dense, thick layer of peptidoglycan, a mesh-like structure composed of alternating β-1,4-linked N-acetylmuramic acid and N-acetylglucosamine chains which are covalently linked by peptide bridges. This dense network serves to provide resistance to environmental stresses and is critical in defining and maintaining cellular integrity and morphology. Ovococcal species, including the ellipsoid lactococci, streptococci and enterococci, are primarily differentiated from the ‘true’, spherical, cocci represented by staphylococci, micrococci and pediococci, among others, by the presence of an equatorial ring of peptidoglycan outgrowth, which surrounds the cell following peripheral peptidoglycan synthesis (Fig. 1), a mechanism which is not observed in true coccoid species [1], [2], [3]. Peptidoglycan acts as a scaffold for various secondary cell wall glycopolymers which decorate the exposed surface of Gram-positive cells. Comprising approximately 50–60 % of the dry weight of the cell wall [4], [5], glycopolymers such as wall teichoic acid, certain capsular polysaccharides (including those of *Streptococcus pneumoniae* which are synthesised via the Wzx/Wzy pathway, and not those utilising a synthase-dependent manner [6]) and rhamnose (Rha)-containing polysaccharides have been shown to be covalently bound to the peptidoglycan layer through the activity of LytR-CspA-Pst (LCP) transferases [7], [8]. In ovococcal species the Rha-containing cell wall polysaccharide (Rha cwps) mediates virulence, host adhesion, immune evasion, phage adsorption and/or antimicrobial resistance, and appears, at least in some cases, to represent a functional replacement for wall teichoic acid (WTA) [9]. Despite the wealth of informaton pertaining to the physiological function of rhamnose-containing cell wall polymers, the genomic loci which encode the enzymatic machinery for their biosynthesis have not been investigated with the same vigour. A comprehensive review of the Rha-containing polysaccharides of Gram-positive bacteria by Mistou and colleagues highlighted the general aspects of Rha cwps biosynthesis and their physiological functions [9]. However, significant insights regarding the relationship between genomic loci, structural composition and architecture, and biosynthesis and physiological function(s) of the ovococcal Rha cwps have since been established [10], [11], [12]. In the current review we will focus on the genome to structure relationship of the Rha cwps of key ovococcal species – streptococci, enterococci and lactococci. We highlight the utility of gene annotation and locus architecture in identifying common elements of the biosynthetic machinery across these clinically and economically important ovococcal species with implications for human and animal disease (streptococci and enterococci) and global dairy fermentation processes (lactococci and dairy streptococci).

### 1.1 Rhamnose-containing cell wall polysaccharides of ovococcoid Gram-positive bacteria

(i) Streptococci

**Fig. 2:**
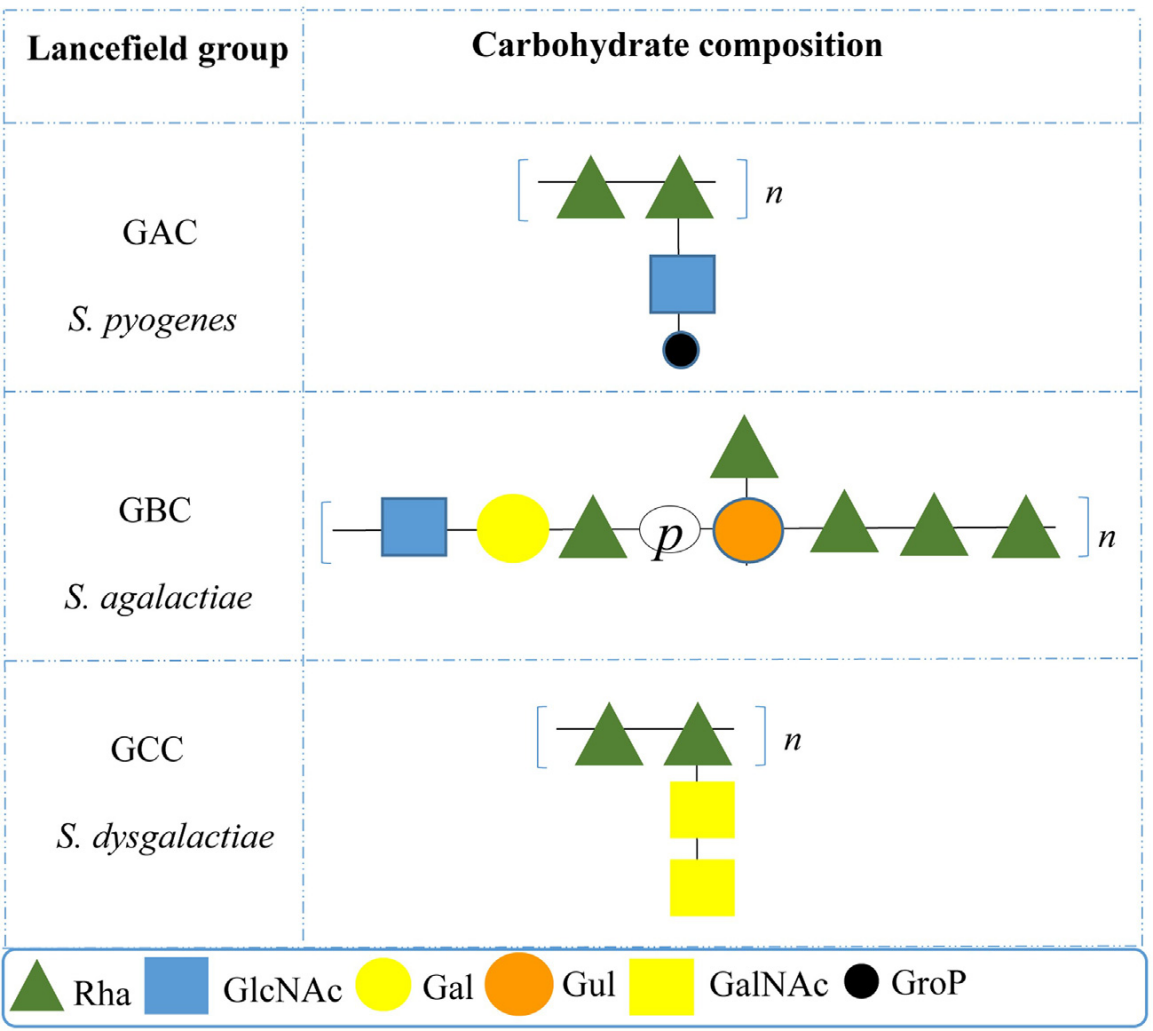
Schematic representation of the biochemical structures of the group specific carbohydrate antigens from select Lancefield streptococci. Adapted from Zorzoli *et al*., [29]. Monosaccharide symbols used are based upon the standard Symbol Nomenclature for Glycans (SNFG) [30].

Species of the genus *Streptococcus* are extensively studied with respect to their cwps structure (and antigenicity) owing to their pathogenesis towards both humans and animals [13], [14]. Streptococcal species may be divided into one of two primary groups, the β-haemolytic pyogenic group or the α-haemolytic viridans group [15]. Seminal works by Lancefield [16], [17] facilitated the classification of β-haemolytic streptococci based on the presence of antigenic, cell surface-exposed carbohydrate groups (designated groups A through V) [18]. Early studies on the streptococcal antigen identified rhamnose as a commonly occurring monosaccharide with glucose (Glc), galactose (Gal), glucosamine (GlcN) also reported in many cases [19], [20], [21], [22], [23]. Furthermore, a common core polysaccharide structure, composed of α1,2- and α1,3-linked rhamnose units was detected in various streptococcal species [24]. As structural and compositional knowledge accumulated, the desire to identify the biosynthetic machinery for this apparently ubiquitous streptococcal polymer increased. A schematic overview of the biochemical structures of the Group specific antigen from selected Lancefield streptococci, including GAC, is presented in Fig. 2.

The non-Lancefield type species *Streptococcus mutans* emerged as the prototype for such investigations with four serotypes (c, e, f, or k) identified based on the site specific linkage of a glucose side chain on the α-1,2-/α-1,3-linked rhamnosyl core of the *S. mutans* Rha cwps [25], [26], [27]. Genes associated with Rha cwps synthesis in streptococci were first identified by Tsukioka *et al.* in *S. mutans* through the functional characterisation of a three gene locus, the products of which are involved in the biosynthesis of the nucleotide precursor sugar dTDP-L-rhamnose [28]. The identified locus, comprised of *rmlA*, *C* and *B,* is directly involved in the formation of the *S. mutans* serotype c antigen, is ubiquitous among streptococcal species and is indispensable for completion of the dTDP-L-rhamnose biosynthetic pathway, and thus by extension, the sero-specific surface antigen [28], [31], [32]. Furthermore, a homolog of *rmlD*, a fourth gene involved in dTDP-L-rhamnose production, was identified in a separate locus, designated *rgp*, which was also shown to be required for Rha cwps biosynthesis. The *S. mutans* rhamnose-glucose polysaccharide (*rgp*) biosynthesis locus is comprised of six core genes*, rgpA* through to *rgpF,* which are essential for Rha cwps synthesis [33]. Disruption of particular genes in the *rgp* locus was shown to cause a reduction in both rhamnose and glucose content of cwps fractions of corresponding mutant strains. Additionally, mutations in *rgpE* abolished glucose side chain addition to the α1,2/α1,3-linked rhamnosyl core [33]. A model for the assembly pathway was proposed for the polyrhamnose structure [24] in which RpgA acts as the initiating rhamnosyltransferase, adding a single Rha residue to N-acetylglucosamine-PP-undecaprenyl (GlcNAc-PP-Und) [9], [29]. Subsequently, RgpB and RgpF are proposed to extend and polymerise the rhamnan chain through the step-wise addition of further rhamnose moieties; RgpC and RgpD, which display homology to known ABC transporter systems, then transport the complete polyrhamnose glycan across the membrane [24]. The *rpgA-F* gene cluster was later found to be conserved between strains regardless of serotype, which is reflective of the conserved α-1,2/α-1,3-linked rhamnan produced by these strains [34]. Beyond the conserved genes of this *rgp* cluster, a single, unconnected gene was found to initiate synthesis of the rhamnan core [35]. Named *rgpG*, the gene was found to complement the UDP-GlcNAc:Und-P-GlcNAc-1-P-transferase activity of WecA, an integral membrane protein which initiates biosynthesis of the enterobacterial common antigen and O-antigen lipopolysaccharide in *Escherichia coli* [35], [36]. The latter finding suggests that the *rgpG* gene product transfers a GlcNAc moiety to the undecaprenyl lipid carrier as the initiating step in rhamnan synthesis. BlastP analysis revealed 62 % amino acid similarity between RpgG and TagO, and deletion mutants of RpgG were found to not only affect Rha cwps synthesis, but to induce morphological defects, altered cell division and changes in biofilm formation [37], [38].

**Fig. 3:**
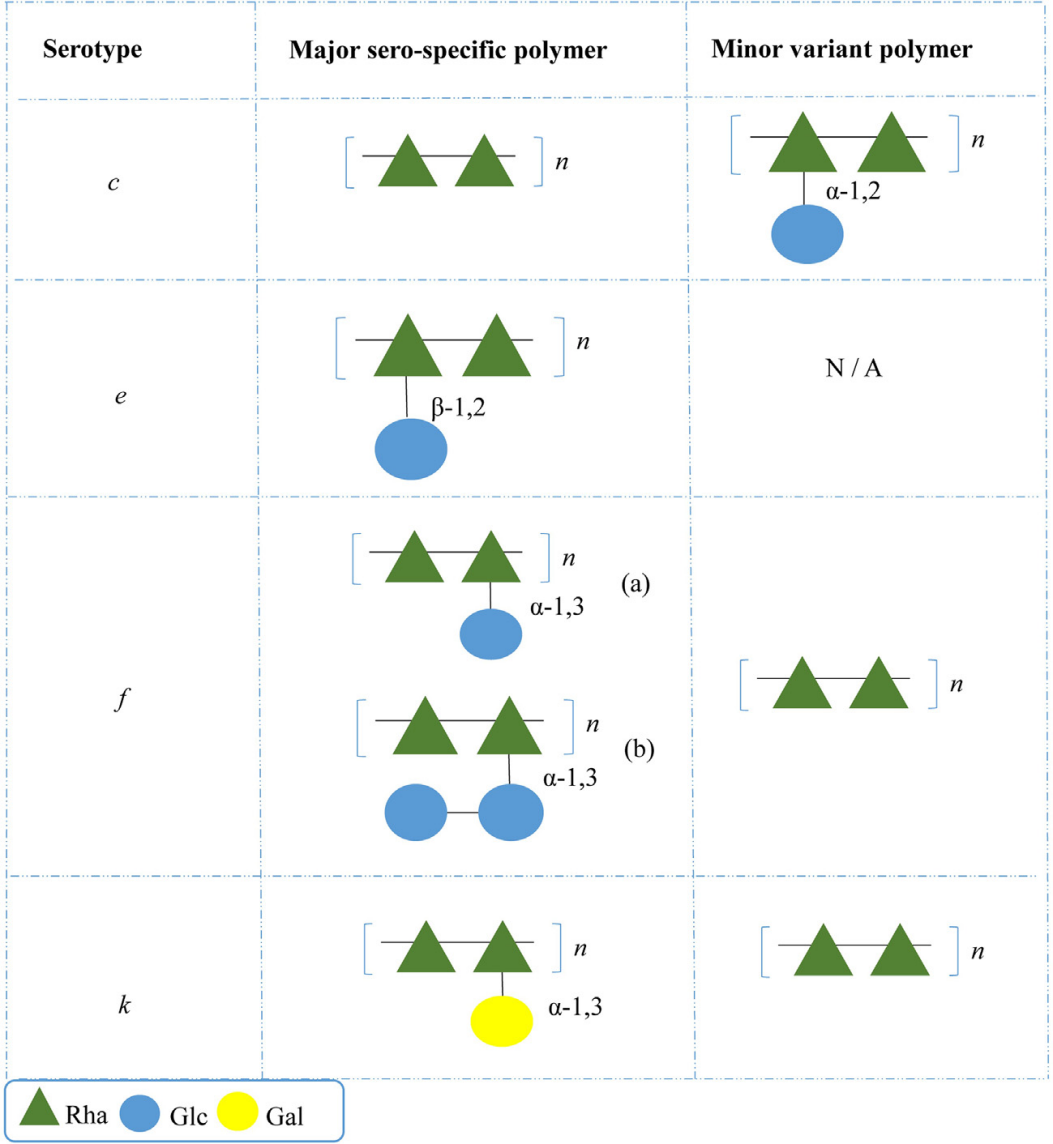
Schematic representation of the *S. mutans* sero-specific antigen major and minor polymers. The position of the Glc/Gal side chain linkage is highlighted. Adapted from St Michael *et al*., [42] and Nakano *et al*., [49]. Monosaccharide symbols used are based upon the standard Symbol Nomenclature for Glycans (SNFG) [30].

The glucose substitution of the *S. mutans* Rha cwps core structure is achieved by means of a number of distinct enzymatic steps. GluA, a glucose-1-phosphate uridylyltransferase, is responsible for the production of UPD-ᴅ-glucose, which then acts as a substrate for RgpE [39]. Ozaki *et al*. extended the boundary the *rgpA-F* locus to include three further genes immediately downstream of *rgpF*. The products of two of these genes, named *rgpH* and *rgpI,* harbour glycosyltransferase domains while it was also determined that RgpH, being similar to RgpE, is required for glucose side chain formation, whilst RgpI alters the kinetics of RgpH, thus controlling branching frequency [26], [40]. The third gene, *orf7*, was later determined to encode a glycerol phosphate transferase which contributes to modification of the Glc side chain structure [41]. The region downstream of *rgpF*, which as mentioned above is responsible for glucose side chain formation, differs significantly between *S. mutans* serotypes c, e and f [34]. Through serotype conversion studies it was confirmed that this variable region mediates the sero-specificity of *S. mutans* by dictating the presence and position of the glucose linkage [34]. More recent structural analysis of the Rha cwps from *S. mutans* serotypes c and f has revealed an unexpected fluidity of the structure by identifying minor and major variant polymers for each type [42] (Fig. 3).

These seminal investigations of the biosynthetic assembly of the *S. mutans* Rha cwps have facilitated the unravelling of equivalent pathways in Lancefield streptococci. For example, genes homologous to *rgpG*, *rmlACBD*, *rgpA* and *rgpB* were identified within the genome of *Streptococcus agalactiae*, a neonate pathogen which expresses the group B carbohydrate (GBC) antigen [43]. The identification of such genes allowed a preliminary assembly pathway to be proposed for the highly complex, multi-antenna structure, which is composed of Rha, galactose (Gal), GlcNAc and glucitol residues, linked by phosphodiester bonds [43], [44]. *S. agalactiae*, was also found to harbour a gene encoding for a TagO/RgpG like protein named GbcO, which has been shown to initiate GBC synthesis by the transfer of a GlcNAc-P moiety to Und-P [45]*.* Data pertaining to the biosynthesis of group C carbohydrate (GCC) antigen is limited, though a locus with 65% identity to the conserved *rpgA-F* region of *S. mutans* was identified in *Streptococcus dysgalactiae* subsp. *equisimilis* 167 [46]. Cell wall fractions of *S. dysgalactiae* 2023, a clinical bovine isolate, possess two distinct rhamnose rich polysaccharides termed RRP1 and RRP2. The branched RRP1 is a GCC, while RRP2 is a linear rhamnose-containing polysaccharide [47]. Similarly, the bovine mastitis-causing strain *Streptococcus uberis* 233 possesses two distinct rhamnan structures: a glucose-containing branched rhamnan and an unbranched, linear version [48].

The Group A carbohydrate (GAC) expressed by *Streptococcus pyogenes* has received significant research attention. The GAC structure consists of the α-1,2/α-1,3-linked rhamnan core which is substituted with GlcNAc moieties. As with *S. mutans*, the GlcNAc substitutions seem to be crucial for the immunogenic response [50], [51]. Van Sorge and colleagues identified the genomic locus which encodes the biosynthetic machinery of the GAC structure based on its homology to genes associated with Rha cwps synthesis [52]. This locus encompasses twelve genes, *gacA-L,* and was functionally annotated by mutational analysis. Three predicted rhamnosyltransferase-encoding genes, *gacA*, *gacB* and *gacC* were recalcitrant to disruption and deemed essential for GAC synthesis and viability. Disruption of *gacI*, predicted to encode a glycosyltransferase, *gacJ*, encoding a membrane protein, and *gacK,* which encodes a Wzx-like transporter [9]*,* resulted in a loss of the GlcNAc-mediated immune response, indicating their involvement in the incorporation of the GlcNAc substitutions [52]. Furthermore, a *tarO*/*gbcO* homolog, termed *gacO* was identified outside of the Rha *cwps* biosynthesis cluster [52]. Experimental evidence suggests that GacO initiates GAC synthesis through the formation of GlcNAc-P-P-Und, which acts as an acceptor for the first rhamnose moiety of the polyrhamnose core [51]. Further insights into GAC synthesis followed, in which detailed characterisation of the *gac* genes allowed for the proposition of an assembly pathway [51]. In a similar architecture to that of the *S. mutans* Rha *cwps* locus, the leftward end of the *gac* locus is flanked by an *rmlD* homolog, *gacA* [32], [51], while the downstream rhamnosyltransferase-encoding genes *gacB*, *gacC* and *gacG* represent functional equivalents of *rgpA*, *rgpB* and *rgpF,* respectively. The ABC-type transport system involved in the export of the polyrhamnose core structure is encoded by *gacD* and *gacE* [51]. Notably, the homolog of *gacB* from *S. mutans* was found to functionally substitute GacB and restore Rha cwps synthesis during heterologous expression and complementation studies in *E. coli* harbouring a *gacA-G;*Δ *gacB* expressing plasmid, indicating a common initiating step for Rha cwps biosynthesis for species within this genus [29]. Investigations into the enzymatic function of genes associated with GlcNAc side chain formation, *gacI*, *gacJ* and *gacL* determined that GacI harbours GlcNAc-P-Und synthase activity which is enhanced by the membrane protein GacJ. Disruption of the polytopic membrane protein GacL was found to result in an intracellular accumulation of the biosynthetic intermediate β-GlcNAc-P-Und and significant reduction in the amount of GlcNAc in cell wall fractions. It was therefore proposed that GacL transfers a GlcNAc residue to the rhamnose core from GlcNAc-P-Und, the biosynthetic intermediate generated through the activity of GacI/GacJ complex [51]. A subsequent study detected GacH-mediated glycerophosphate modification of the GlcNAc side chain moiety of GAC at a frequency of approximately 25 % [41].

Although rarely discussed in the realm of streptococci, likely due to its non-pathogenic status, the dairy-associated species *Streptococcus thermophilus* also harbours homologs of genes relating to Rha cwps synthesis, including those of the well characterised *S. mutans rgpA-F* core synthesis genes [53]. However, as shown by Hols *et al*. the genetic arrangement and architecture of the cluster is distinct from other species of this genus, as the genes encoding the variable or side-chain structures of the *S. thermophilus* Rha cwps precede those encoding the core, which are represented by *rgpA*-*F* homologs [53]. A presence/absence-based hierarchical clustering of the Rha *cwps* locus of twenty three industrial strains of *S. thermophilus* revealed a high level of genetic diversity allowing the assignment of five distinct Rha cwps- associated genotypes, designated A-E [54]. This analysis was expanded by Romero and colleagues to include the Rha cwps locus from *S. thermophilus* genomes available through NCBI databases in addition to proprietary strains [55]. The boundaries of the locus were also extended with *rpoD* and *radC* serving as the relevant start and end flanking genes, noting that these genes play no role in Rha cwps synthesis. 167 strains were found to cluster into one of three major groups (A – C). Group A contains seven subgroups (A_1-7_), Group B contains six subgroups (B_1-6_) and Group C, five subgroups (C_1-5_), with former Groups D and E now represented by A_7_ and C_4,_ respectively. Furthermore, *rgpF* was found to be a genotypic marker for the major groups based on inter- and intra-group sequence identity. A second group-specific attribute was the lack of an *rgpE* equivalent within strains classified as Group B [55]. Although preliminary functional annotations have been made for genes within the cluster, the overall gene content of the *S. thermophilus* Rha *cwps* clusters remains poorly characterised with limited experimental proof of the associated protein functions. Interestingly, investigations into the role of the two component signal transduction systems (TCS) TCS06 and TSC07 of *S. thermophilus* LMD-9 in response to bacitracin exposure, indicated that the response regulator of TCS06, encoded by *rr06,* acts as a transcriptional repressor of *rpgA*, *rgpB* and *rgpC*, and as a transcriptional activator of *rgpI*. The response regulator of TSC07, encoded by *rr07*, is hypothesised to repress transcription of *rmlC*. Taken together, these results suggest a functional role for *S. thermophilus* Rha cwps as an antimicrobial barrier [56]. Furthermore, *tagO* was implicated in an increased acquisition of the integrated conjugative element ICESt3 [57]. This suggests a critical, functional role for TagO in maintaining cell wall integrity in *S. thermophilus,* akin to its pathogenic counterparts. A preliminary pathway for Rha cwps core structure biosynthesis in *S. thermophilus* LMD-9, based on the *S. mutans* model, has been proposed by Thevenard *et al*. [56].

Compositional analysis of the monosaccharide content of the cell walls from the *S. thermophilus* strains STCH_12 and STCH_15 revealed the presence of rhamnose, galactose and glucose [58]. However, the biochemical structure of an *S. thermophilus* Rha cwps has been elucidated for just a single strain, i.e. ST64987 [59]. The complex structure is composed of a repeating tetrasaccharide core, which is composed of rhamnose and glucose moieties, and which is decorated with tri- and tetra-saccharide side-chains, that possess GlcNAc at their branching points [59].

Overall, Rha cwps synthesis in streptococci is dependent on the presence of the core *rgpA-F* encoded rhamnosyltransferases in addition to the UDP-GlcNAc:Und-P-GlcNAc-1-P-transferase activity encoded by a *tagO* homolog. The individual, strain/species-specific biological characteristics of the Rha cwps structure are underpinned by the diversity of glycosyltransferases encoded by the variable regions of the Rha cwps-associated loci.

(ii) *Lactococcus lactis*

*Lactococcus lactis* is the most extensively applied Lactic Acid Bacterial (LAB) species in commercial and artisanal dairy fermentations. Four subspecies have been characterised – *L. lactis* subsp. *lactis*, *L. lactis* subsp. *cremoris*, *L. lactis* subsp. *hordniae* and *L. lactis* subsp. *tructae* – with subsp. *lactis* and subsp. *cremoris* representing the most important subspecies from a biotechnological perspective [60], [61], [62]. *L. lactis* has been subject of focused research attention in recent decades, not only for its ability to impact on the rheological and organoleptic properties of fermented products, for example through plasmid-encoded exopolysaccharide production [63], [64], but also for its ability to produce antimicrobial compounds such as nisin, a bacteriocin utilised in food preservation [65], [66]. Furthermore, it has become a model Gram-positive organism for the study of phage-host interactions [67], [68], [69].

The Rha cwps of lactococci are comprised of two, distinctly synthesised saccharidic components – a highly conserved, neutral, peptidoglycan embedded di- or tri-saccharide rhamnan (Rha) and a surface-exposed decoration (previously termed the polysaccharide pellicle (PSP)) which exhibits a high degree of structural variability [10], [12], [70], [71]. The observed structural flexibility of lactococcal Rha cwps is believed to be driven by a need to protect and respond to extra-cellular pressures, in particular phage predation. As such, lactococcal Rha cwps structures appear to be in a state of constant evolution, leading to the emergence of novel features and ultimately divergent cwps groups [10].

The genes responsible for lactococcal Rha cwps biosynthesis are clustered within a single locus, first identified by Dupont *et al*. in *L. lactis* subsp. *lactis* IL1403 and *L. lactis* subsp. *cremoris* Wg2 through transposon mutagenesis [72]. The leftward end of the lactococcal Rha *cwps* locus is highly conserved and encompasses genes encoding the enzymatic machinery for the biosynthesis of the rhamnan component including *rmlA-D* and *rgpA-F,* in addition to a lactococcal-specific gene, *wpsJ*. WpsJ is implicated in the transfer of the decorative element to the rhamnan core [10], [12]. *In silico* characterisation of genes located in the 5′ region allowed the proposition of a model for Rha cwps biosynthesis in *L. lactis*. In keeping with secondary cell wall polysaccharides of other species [9], a TagO ortholog (llmg_1976) is predicted to initiate rhamnan biosynthesis through its encoded UDP-GlcNAc:Und-P GlcNAc-1-P transferase activity; dTDP-L-Rha is synthesised by the enzymatic activity of the *rmlACBD* gene products; the poly Rha chain is built on the lipid-linked GlcNAc moiety via RgpA, RgpB and RgpF and transported across the membrane by ABC transporter system proteins RgpC and RgpD*.* Covalent incorporation of the Rha polymer into the peptidoglycan layer is believed to be a function of the LcpA ligase [71].

**Fig. 4:**
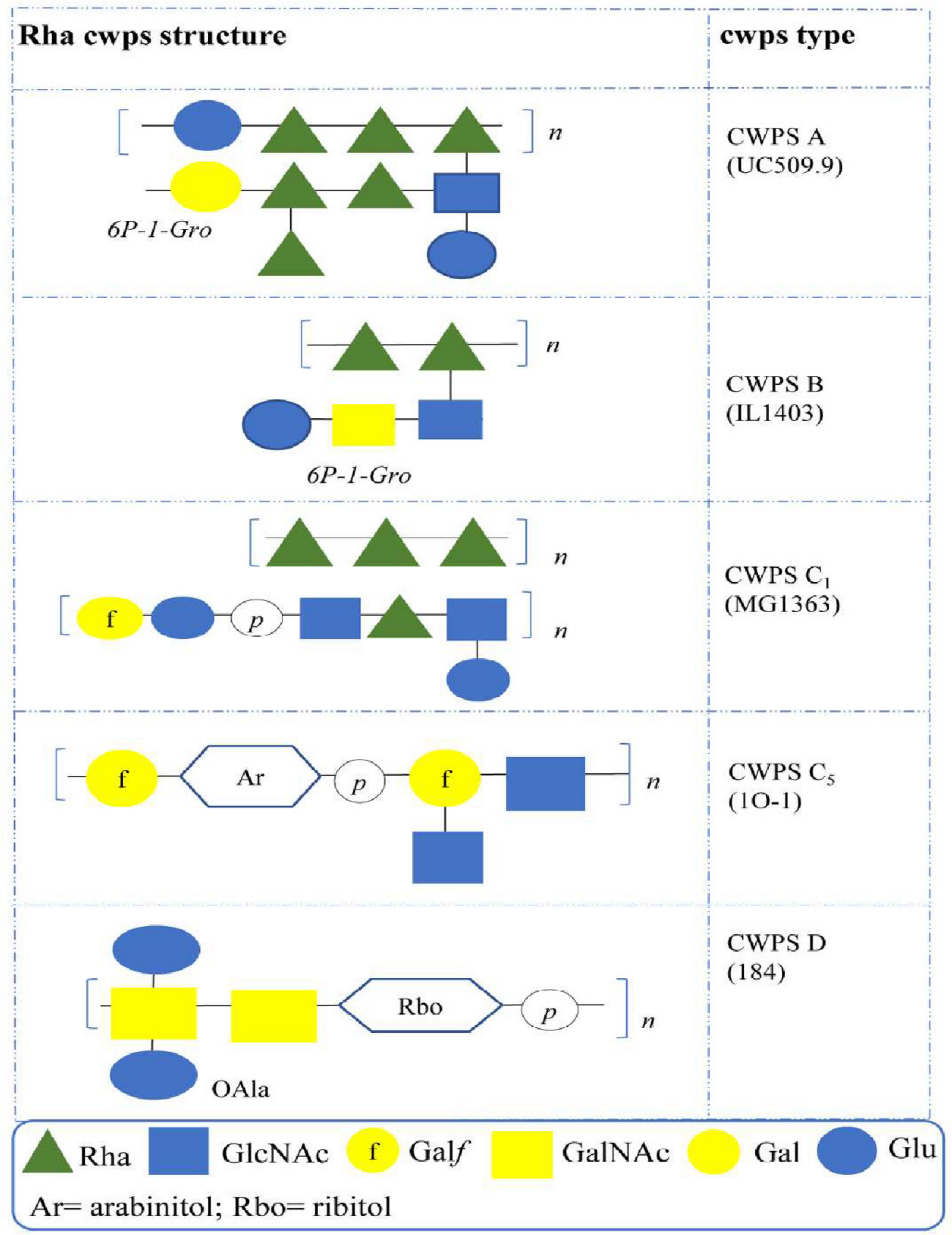
An overview of the variation observed in the biochemical structure of the *L. lactis cwps* types. Adapted from Mahony *et al.,*[10]. The rhamnan structures for *L. lactis* 1O-1 and *L. lactis* 184 have not yet been elucidated. In the case of *L. lactis* MG1363, the nature and site of the linkage between the rhamnan core and decorative side chain has not yet been defined. Monosaccharide symbols used are based upon the standard Symbol Nomenclature for Glycans (SNFG) [30].

The rightward (or 3′) end of the locus displays a high level of diversity among different lactococcal strains and encodes the biosynthetic machinery for production of the decorative element or side chain of the Rha cwps [10], [73]. The diversity of this region facilitated an initial classification of lactococcal strains into one of three major *cwps* genotypes – A, B or C [74]. Later studies introduced further diversity through the identification of C type subgroups C_1-8_ and the novel group D [10], [73]. Advances in the structure–function relationship of genes involved in Rha cwps biosynthesis prompted a comparative investigation into the Rha *cwps* loci of 107 lactococcal strains. The genotypic characterisation of the lactococcal Rha *cwps* clusters is defined by the variable glycosyltransferase-encoding genetic content of the 3′ region of the *cwps* locus [10], [73]. For example, genotypes A and B lack synteny in the 3′ region, yet both harbour genes encoding functions related to the incorporation of GroP residues and a TagD1-like cytidylyltransferase, which are absent among genotype C strains [10]. The intergroup diversity of genotype C subgroups is limited and may be based upon the presence/absence of a single gene, transposase interruption or gene truncation [10], [73]. Genotype D, represented by *L. lactis* strain 184, is completely distinct and is predicted to encode unique nucleotidyltransferases and a single gene encoding an alcohol dehydrogenase. The elucidation of the biochemical structures of the Rha cwps fractions from seven strains representing C type subgroups C_1_, C_3_, C_4_, C_5_ and C_6_ in addition to the archetypal genotype D strain further confirmed that the genetic complexity of the side chain-specifying region of the Rha cwps-associated loci is reflected in associated biochemical architectures (Fig. 4) [10]. Furthermore, by underscoring the close correlation between *cwps* genotype and the chemical structure of the associated polysaccharide the incorporation of specific/modified moieties such as Gal*f* or glycerophosphate can now be predicted with confidence [10].

A dual chain assembly pathway, encompassing the complete Rha cwps (i.e. Rhamnan polymer plus side chain) of *L. lactis* MG1363 has recently been proposed [12]. Decorative side chain synthesis is initiated by the priming glycosyltransferase, WpsA and its associated membrane protein WpsB, through the addition of a GlcNAc moiety to Und-P. Cytosolic glycosyltransferases WpsC, WpsD, WpsE and WpsF elongate the decorative subunit, which is subsequently transported across the membrane by the Wzx-like flippase, WpsG. Polymerisation of the subunits is a function of the polymerase WpsI and the co-polymerase WpsH, while WpsJ completes the synthesis pathway though the covalent attachment of the decorative subunits to the peptidoglycan-linked rhamnan [10], [12]. Mutational analysis of genes encoding decorative side chain synthetic functions provided experimental support for the proposed pathway with biochemical analysis of *wps* gene mutants further corroborating these findings [12].

The side chain subunit structures of distinct strains may be of varying length, while the subunit structures may also be polymerised in some, but not all, cases [10]. Furthermore, the combination and architecture of the component (oligo)saccharides of the Rha cwps is linked to the high degree of specificity of lactococcal phages (this will be discussed in more detail later). Indeed, the rhamnan and side chain structures may each be further decorated through the activity of so-called three component glycosylation systems (TGS) that are encoded by genes located outside the *cwps* gene cluster [75]*.* Functional characterisation of the gene pairs – *csdAB* and *csdCD,* along with their associated flippase-encoding gene, *cflA,* determined that they are involved in the glycosylation of rhamnan core and the side chain, respectively. A review of 33 publicly available genomes indicated that *csdAB* and *csdCD* are present in nine and ten strains, respectively. The detection of additional genomic loci in particular lactococcal strains, which modify components of the Rha cwps, underpins possibilities for their structural diversification. It also highlights that these modifications are not universal and may be a response to external and environmental pressures [75].

(iii) *Enterococcus*

The genus *Enterococcus* was first defined in 1984 following DNA-DNA hybridisation studies, which showed that certain species of enteric streptococci (then named *Streptococcus faecalis* and *Streptococcus faecium*) were distantly related to non-enteric streptococci [76]. Although a commensal species in the gut microbiome of mammals and birds, enterococci can also be found in a variety of environmental niches [77]. In recent years, enterococci have gained attention due to the emergence of *Enterococcus faecalis* and *Enterococcus faecium* as rapidly evolving opportunistic pathogens, particularly in nosocomial environments [78]. The cell surface of enterococci possesses multiple, secondary glycopolymers [79], including lipoteichoic acids (LTAs), wall teichoic acids (WTAs), variably present capsular polysaccharides (CPS) and the ubiquitous enterococcal polysaccharide antigen (EPA), which represents the Rha cwps for this species. The EPA is encoded by a large 40.6 kb cluster of genes arranged in two component modules: the conserved *epaA-R* region located at the 5′ end, which is involved in the synthesis of the rhamnose-containing EPA core polysaccharide and the 3′ variable region which harbours various glycosyltransferases, epimerase/dehydratases and membrane proteins which encode the biosynthetic machinery for EPA glycan decorations [11].

The presence of an antigenic polysaccharide was first confirmed through the screening of genomic libraries of *E. faecalis* OG1RF and TX52 against patient sera and sera generated from rabbits immunised with *E. faecalis* surface proteins. Seven of the generated cosmid clones were found to only react to patient sera and a representative clone, BO-4B61 was insensitive to proteinase K treatment, suggesting the presence of a non-protein antigen such as Rha cwps [80]. Production of the saccharidic antigen was found to be associated with a gene cluster of 43 kb harbouring genes associated with polysaccharide biosynthesis, including the conserved *rml* operon [9], [81], [82]. Further characterisation defined the region as *epaA* to *epaR* and compositional analysis of purified EPA from *E. faecalis* OG1RF confirmed the presence of Glc, Rha, Gal, GlcNAc and N-acetylgalactosamine (GalNAc) residues [83]. Conservation of the *epaA-R* locus across *E. faecalis* strains was confirmed by hybridisation studies, while variation was observed in the region downstream of *epaR* between the clinical, vancomycin resistant isolate V583 and OG1RF and in the architecture of the cluster [83], [84]. An *epa* locus harbouring a similar downstream extension of the *epaR* region was also noted in *E. faecium* TX16. The overall architecture of the core genes was found to differ from the latter *E. faecalis* strain in that *epaI, epaJ* and *epaK* were absent, being apparently substituted by *epaP* and *epaQ* [85]. Palmer *et al*. exposed the extent of variation present within the *epa* loci of both *E. faecalis* and *E. faecium* through a comparative genomics study of enterococcal species [86]. Notably, WTA synthesis-associated genes, *tagF,* which encodes a teichoic acid polymerase*,* and *tagD*, encoding a cytidylyltransferase, which provides activated phosphate for GroP synthesis, were identified within the variable region of both *epa* loci of *E. faecalis* and *E. faecium* [86]. For a comprehensive schematic overview of *epa* locus diversity in *E. faecalis* and *E. faecium*, see Palmer *et al*. [86].

Further divergence of the *epa* locus was found in both commensal and pathogenic strains of *Enterococcus cecorum*, a bacterium which causes outbreaks of enterococcal spondylitis (ES), a severe disease in broiler chickens characterised by hind limb paresis and paralysis and incidences of hepatitis and pericarditis [87], [88]. Here, *epaA* is genetically remote from the *epa* cluster. The core *epa* locus for this species is therefore comprised of *epaB-H* and shares a high level of identity with that of V583. The region downstream of *epaH* is highly divergent between pathogenic and commensal strains in terms of genetic content and architecture, which is consistent with the variable region of other *epa* loci [88].

**Fig. 5:**
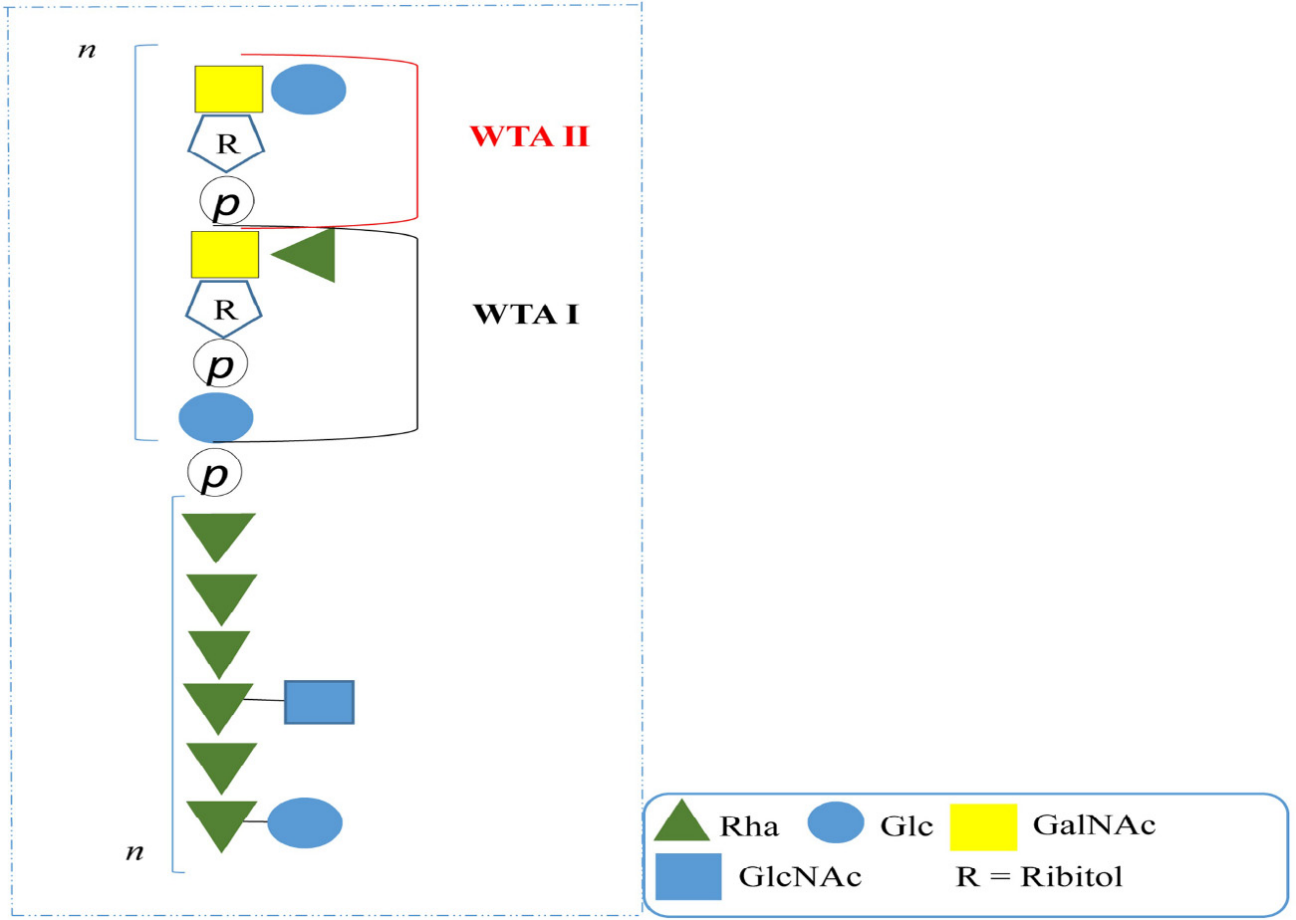
Schematic representation of the complex biochemical structure of the *E. faecalis* V583 EPA in which WTAs are stacked onto the polyrhamnose core. Adapted from Guerardel *et al*., [11]. Monosaccharide symbols used are based upon the standard Symbol Nomenclature for Glycans (SNFG) [30].

Numerous mutational studies have confirmed that the EPA affects multiple processes including biofilm formation, virulence, host colonisation and immune evasion, conjugative transfer, phage resistance, cell wall integrity, cellular morphology and antimicrobial resistance [83], [89], [90], [91], [92], [93], [94]. Despite the wealth of knowledge pertaining to the biological functions of EPA, its biochemical structure was not elucidated until 2020 [11]. Detailed NMR analysis of the isolated cell wall fractions of *E. faecalis* VE14089, a plasmid-cured derivative of V583 revealed a highly complex polymer composed of a αGlc- and βGlcNAc-substituted rhamnan core onto which teichoic acid decorations are attached by phosphodiester linkages (Fig. 5). Remarkably, the NMR and structural data relating to the teichoic acid decorations were found to be identical to those previously described by Geis *et al*. as WTA Ⅰ and WTA Ⅱ [95]. Interruption of *tagB,* a glycerophosphate transferase-encoding gene, induced compositional changes to the EPA [95], [96] highlighting the interconnected relationship between these secondary glycopolymer structures. Since both WTAs form part of the mature EPA structure, it is logical that Δ*tagB* mutants not only abolish WTA Ⅰ and WTA Ⅱ from the cell surface, but also induced compositional changes in the overall Rha cwps [95]. Of note, both WTA decorations were not detected in an isogenic Δ*epaX* mutant which is hypothesised to transfer a GalNAc residue to the WTA structure during assembly [11], [97]. Additional analysis of whole cells demonstrated that while the WTA decorations are flexible and surface-exposed, the polyrhamnose core is embedded within the cell wall with minimal exposure [11]. Using functional prediction, it has been proposed that the polyrhamnose core is synthesised internally and transported across the membrane by an ABC-type transport system, after which it is modified by the addition of Glc and GlcNAc residues. The WTA decorations are synthesised and modified independently before being linked and transported as a single unit via a Wzx/Wzy pathway. Attachment to the rhamnan core and anchoring to the peptidoglycan layer is believed to be a function of one of five LCP proteins encoded by *ef0465*, *ef1212*, *ef1569*, *ef2703* and *ef3245*, which are located outside of the *epa* locus, thus producing a mature EPA [11]. Very recently, the truncation of *galU,* a UDP-glucose-1-phosphate uridylyltransferase-encoding gene, has been shown to result in loss of EPA production due to UDP-glucose depletion [98].

### 1.2 Shared features of cell wall polysaccharide biosynthesis in ovococcoid bacteria

**Fig. 6:**
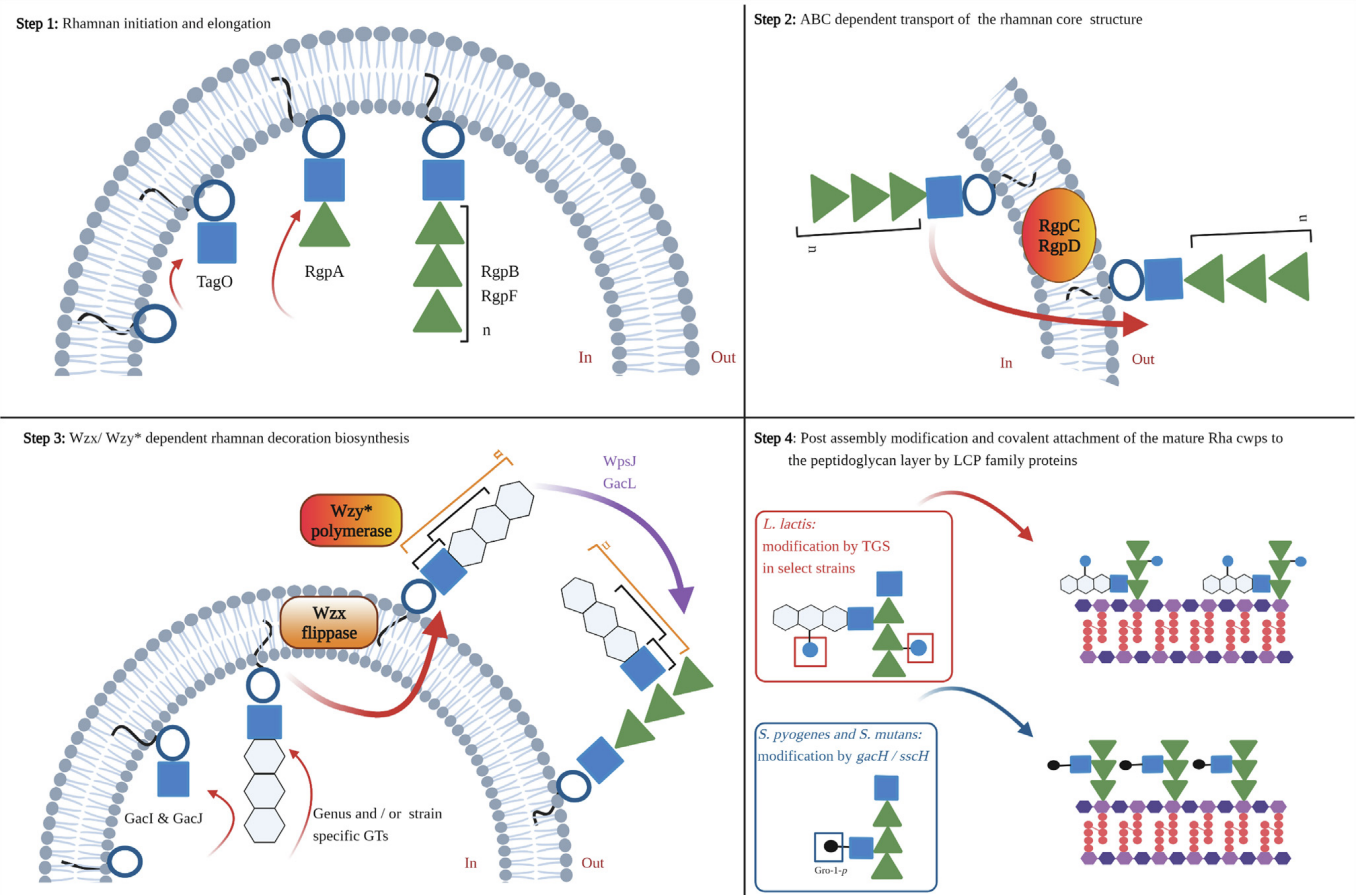
Overview of Rha cwps biosynthesis of ovococcal species. **Step 1**: synthesis of the rhamnan core is initiated by the transfer of a GlcNAc moiety to the lipid carrier by *TagO* homologs. RgpA adds the first rhamnose and RgpB and F work in tandem to elongate the chain. **Step 2**: The rhamnan core polymer is transported across the membrane by an ABC transporter system encoded by *RgpC* and *RgpD*. **Step 3:** The decorative structures of the rhamnan core are independently initiated by the transfer of a GlcNAc moiety to the lipid carrier though the activity of the *gacI/gacJ* like gene pair. The decorative structures may be mono-saccharidic, di-saccharidic or oligo-saccharidic in nature and their composition is dictated by the gene content of the variable regions of the Rha *cwps* locus. The decorative units are flipped to the outer side of the membrane by a Wzx flippase, and in some cases (such as *L. lactis* MG1363) the individual subunits are externally polymerised by an encoded *Wzy* polymerase. Attachment of the decorative structures to the rhamnan core is believed to be a function of the membrane associated proteins GacL (*S. pyogenes*) or WpsJ (*L. lactis*). **Step 4:** Post assembly modification of the rhamnan decoration may occur and can be a function of proteins encoded within the Rha *cwps* locus, such as GacH which adds Gro-P to the GlcNAc decoration or, of external loci, such as the TGS of select *L. lactis* strains which modify the decorative chain by the addition of a Glc moiety. The final attachment of the mature Rha cwps is believed to be a function of LCP family proteins. Image created with BioRender.com.

The elucidation of biosynthesis pathways pertaining to rhamnose-containing cell wall polysaccharides in ovococcal species has revealed a number of common enzymatic reactions relating to initiation, elongation and modification. Similarly, it is evident that a modular Rha cwps-associated locus is present in species which produce a complex polysaccharide structure or highly modified linear rhamnan cores [10], [12], [86]. By focusing on the well characterised Rha cwps biosynthesis pathways of *S. pyogenes* (GAC), *E. faecalis* V583 (EPA) and *L. lactis* NZ9000, several commonalities emerge with reference to locus architecture, gene function and resulting Rha cwps structure. A schematic overview of the generic Rha cwps biosynthetic pathway is presented in Fig. 6 and specific aspects are discussed further below. Table 1 further highlights the key proteins involved in the synthesis of both the rhamnan core and their respective decorative side chain structures.

(i) Rhamnan core biosynthesis

**Table 1:** Overview of the key proteins involved in Rha cwps biosynthesis from well characterised ovococcal genera.

Rhamnan core					
Genus	Initation	1st Rha	Transport	Polymerisation	Attachment
*Streptococcus*	RgpG GbcO GacO	RgpA GacB	RgpC/D GacD/E	Rgp B/F	Probable LCP family
*Enterococcus*	EpaA	EpaB	Epa L/M	EpaC/D/O/N	LPC family
*Lactococcus*	TagO	RgpA	RgpC/D	RgpB/F	LCP family

Rhamnan decoration					
Genus	Initiation	Elongation	Transport	Polymerisation	Attachment
*Streptococcus*	GacI/J	N/A	GacK	N/A	GacL
*Enterococcus*	EpaI/J	GT-2 family	EF2166	EpaY	LCP family
*Lactococcus*	WpsA/B	GT-2 family	WpsG	WpsI/H	WpsJ

Rhamnan polysaccharides are believed to be a functional replacement of WTA [9], the synthesis of which is initiated by the UDP-GlcNAc:Und-P-GlcNAc-1-P transferase activity of TagO [99]. As discussed above, it is now evident that ovococcal species which lack WTA as the major cell wall polymer, have repurposed the function of TagO to initiate synthesis of a Rha-based cwps. Variability exists primarily in the genomic position of the initiating gene. For example, the enterococcal equivalent to *tagO*, embodied by *epaA*, is the first gene of the *E. faecalis epa* locus, yet is remote from the locus in *E. cecorum* [11], [88]. Similarly, the *L. lactis tagO* homolog, and that of multiple streptococcal species, is positioned at a distinct genetic location from that of the primary Rha *cwps* locus [11], [12], [37]. The dTDP-L-rhamnose required for synthesis of the polyrhamnose core is provided by the functions of the *rml* locus [9]. In the case of *L. lactis* and *E. faecalis*, the *rmlA-D* genes are clustered together at the 5′, conserved end of the *cwps* locus. In contrast, a distinct architecture is observed in streptococcal species in which *rmlA, C,* and *B* are located at an unconnected genomic location [9], [11], [12], [29]. Despite these variations, it appears that all species utilise a common pathway for the establishment of a dTDP-L-Rha pool in the early stages of Rha cwps synthesis.

The addition of the primary rhamnose unit to the undecaprenyl-PP-linked GlcNAc foundation is carried out by the first rhamnosyltransferase encoded within the associated Rha cwps-associated locus of each species. For streptococcal Lancefield Groups A, B, C and G, this rhamnosyltransferase is a highly conserved α-D-GlcNAc-β-1,4-L-rhamnosyltransferase [29], whereas in *E. faecalis* and *L. lactis* α-1–3/α-1–2 rhamnosyl transferases are predicted to complete the respective rhamnose transfer [11], [12]. Extension and polymerisation of the rhamnose chain is a function of the remaining glycosyltransferases within the 5′ conserved region of the respective loci and in all cases an ABC-type transporter system is used to transfer the polyrhamnose structure across the membrane [11], [12], [51], [100].

(ii) Rhamnan core decoration

In the case of the *S. pyogenes* GAC, the rhamnan backbone component is modified by the addition of an antigenic GlcNAc moiety. Detailed experimental evidence has demonstrated that the UDP-GlcNAc:Und-P-GlcNAc transferase activity of GacI, aided by the membrane protein GacJ, is required for this modification [51], [100]. Rush *et al*. identified GacI homologs in GBS and *E. faecalis* [51], while a similar protein was identified in *L. lactis* NZ9000 [12]. A review of the gene annotations from both *E. faecalis* and *L. lactis* Rha cwps-associated clusters confirms the presence of a gene encoding a DUF2304 domain, typical of GacJ-like proteins, immediately downstream of the *gacI* homolog [11], [12]. In the case of *E. faecalis*, this gene pair is embodied by *epaI-epaJ,* and akin to their GAC counterparts, their activity produces a lipid-linked GlcNAc moiety which is transferred to the EPA rhamnan core by one of two membrane proteins EpaP or EpaQ [9]. The modifying Glc residue is provided by the activity of distinct genes and not the *epaI-epaJ* gene pair [11]. For *L. lactis* the equivalent gene pair, named *wpsA*-*wpsB*, is present in all known *L. lactis cwps* operons [10] and functions as the initiator of decorative side chain synthesis [12]. Overall, it may be concluded that these species utilise a homologous gene pair to produce a lipid-linked GlcNAc moiety, the subsequent transfer of which is a function of species/strain-specific Rha cwps assembly, wherein the GlcNAc moiety may be directly attached to the rhamnan core or be extended to form longer decorative structures.

### 1.3 Shared features of cell wall polysaccharide biosynthesis in ovococcoid bacteria

**Fig. 7:**
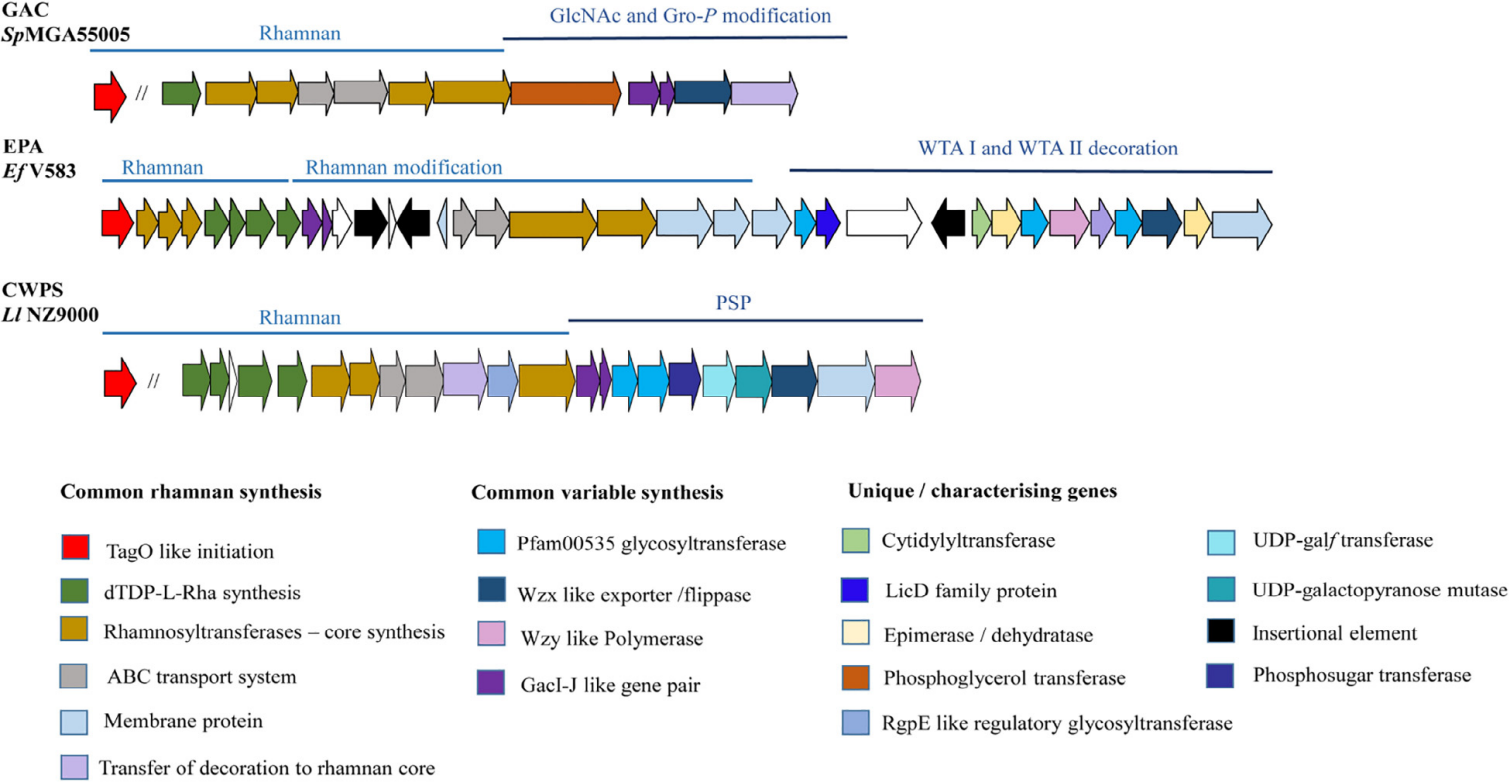
Overview of the modular localisation of genes specific to core rhamnan synthesis and subsequent decoration. The GaI-J like gene pair (purple) are central to the formation of a universal lipid linked GlcNAc foundation from which the polyrhamnose core is decorated. (For interpretation of the references to colour in this figure legend, the reader is referred to the web version of this article.)

For many of the above discussed species, elucidation of the biochemical structures of the Rha cwps is not accompanied by an in-depth characterisation of the variable region of the locus. This substantial knowledge gap leads to difficulty in establishing common structure–function relationships between species with particular reference to those species which produce highly complex rhamnan polysaccharide decorations. Nevertheless, detailed functional assignment of individual genes within the *epa* and *cwps* loci of *E. faecalis* V583 and *L. lactis* NZ9000, respectively, have allowed the identification of common biochemical steps (Fig. 7). For example, the transfer of individual sugar moieties to the growing precursor subunit of the decorative side chain is primarily a function of cytosolic glycosyltransferases containing a PF00535 domain. Furthermore, both systems utilise a Wzx-dependent mechanism for export of the complex decorative structures; in cases where the decorative subunits are polymerised, an encoded Wzy polymerase is employed, and the presence of LicD family proteins is associated with the transfer of Rbo-P units in the case of *E. faecalis* and Gro-P units for *L. lactis* cwps types A and B [10], [11].

Mahony *et al*. [10] have recently shown that the gene content of the variable region of the *L. lactis* Rha *cwps* locus can be used to predict structural features including decoration, composition and polymerisation, and the presence of unique features such as Gro-P or Gal*f* residues [10]. Therefore, by applying this current dogma to additional species, in conjunction with shared functional predictions based upon bioinformatic data analysis and gene annotation, it is now possible to predict various characteristics of the Rha cwps structure, such as the order of their incorporation, the presence of unique modifications, and the presence of oligo- or polysaccharide side chain structures. Informative gene annotations has been a bottleneck for genome-based research for the past two decades; however, such predictive abilities will be transformative to a deeper understanding of the diversity of the associated chemical structures and their implications for strain-specific phenotypes, phage-host interactions among other environmental and industrial considerations. It is well established that phages infecting lactic acid bacteria typically exhibit high host-recognition specificity. The diversity of cwps composition and architecture, the introduction of modifications on both the rhamnan backbone and side chain structures alludes to an external pressure that drives this need for constant modification and diversification. In all ecological niches and in an industrial context in particular, phages are a major driver of host evolution. Below we summarise a number of model phage-host systems in which Rha cwps is defined to mediate phage binding and infection.

### 1.4 Rhamnose cell wall polysaccharides as phage receptors in ovococci

(i) *Streptococcus*

The nature of the host-encoded phage receptor of haemolytic streptococci has not been studied in detail to date. Early studies of phage-host interactions of haemolytic streptococci uncovered a group antigen-specific pattern of infection in which purified Group C antigen was found to neutralise Group C-infecting phages, but not those which infect Group A strains. In contrast, purified Group A carbohydrate was unable to inactivate Group A infecting phages [101] and phage A25, which infects Group A strains is hypothesised to require intact cell wall and peptidoglycan fractions for optimal adsorption [102]. The temperate phage, P9, has been reported to adsorb to the purified Group C antigen of both *S. equi* and *S. zooepidemicus*, yet is unable to infect or replicate on the latter [103], [104]. The Group C antigen has also been identified as the probable receptor for the prototype phage C1 with specific reference to the importance of the GalNAc moiety [105], [106]. In contrast to the above, the choline residues of TA have been identified being pivotal for infection of *S. pneumoniae* by phage Dp-1 [107], [108].

Significant data defining the role of the surface antigen in phage adsorption for the non-haemolytic species *S. mutans* has been provided. Phage M102 is specific to serotype c strains of *S. mutans* and cannot adsorb to serotypes e, f or k, and the Glc side chain of the serotype c antigen has been shown to be critical for both adsorption and infection by this phage [26]. Unequivocal proof was provided through sero-conversion experiments, in which the exchange of a *S. mutans* type e sero-specific locus with that of an *S. mutans* type c locus (identified as the region between *rpgF* and *orf12*) led to a dramatic increase in phage adsorption [26]. In addition, phage M102 is also unable to adsorb to strains that carry mutations in the *rgpA*, *rgpB* or *rgpG* genes [26]. The host-encoded receptor for phages of *S. thermophilus* are also believed to be saccharidic in nature [109], [110]. Preliminary evidence suggests that mutations within a glycosyltransferase encoding gene of the Rha cwps-associated *locus* confers a phage resistance phenotype, yet this remains to be experimentally confirmed [54], [58].

(ii) *Lactococcus*

Early studies of phage-host interactions in *L. lactis* identified rhamnose-containing cell wall components as the receptor for phage kh [111], while mutations within genes of the Rha *cwps* locus of lactococcal strains cause phage insensitivity due to adsorption deficiency as mentioned above. Remarkably, the host range of the prolific 936 lactocococal phage species (now termed the Skunaviruses) and their associated receptor binding phylogeny, can be directly correlated to the Rha *cwps* genotype [74]. Genotype swapping of the C_2_ variable region of *L. lactis* 3107 with that of the C_1_ type strain *L. lactis* NZ9000 confirmed that the locus also mediates infection by the P335 phages ΦLC3 and TP901-1 [73]; however, a direct correlation to host range and Rha *cwps* genotype could not be established for this genetically heterogenous phage species [112]. The less commonly encountered lactococcal phage groups including 949 and P087, have also been shown to recognise moieties encoded by the Rha cwps-associated locus [113], while the *Ceduoviridae* (formerly termed c2 phages) recognise and bind to both an as yet undefined saccharidic component, and a protein (phage infection protein, PIP) receptor on the cell surface [114].

(iii) *Enterococcus*

The EPA has been linked to phage infection in siphophage ΦNPV1, which is unable to form plaques on Δ *epaB*, *M*, *E* and *N* derivatives of the host strain [83]. A later study by Chatterjee *et al*. involving transposon mutants identified the glycosyltransferases *epa*OX and *epa*OX2 of *E. faecalis* OG1RF as critical for adsorption of ΦVPE25 and noted that disruption of the O-antigen ligase, *epa*OY significantly affects plaquing efficiency [115]. Furthermore, an independent transposon mediated disruption of an encoded LytR-type response regulator was found to downregulate transcription of *epa*OX, *epa*OX2 and *epa*OY, resulting in a 40 % reduction of the absorbance of ΦVPE25 [115]. The role of the EPA in host attachment is not limited to *Siphoviridae* phages*.* Adsorption of phage Idefix, which belongs to the *Podoviridae* family, is dependent on *epaX*, encoding a glycosyltransferase which is hypothesised to add a GalNAc moiety to the teichoic acid decorative chain of the EPA [11], [116], whilst infection of myophages EF1TV, phi17 and phi19 also involves EPA-associated genes [117], [118].

Duerkop *et al.* [119] additionally identified a conserved integral membrane protein in *E. faecalis* V583 which is essential for infection by siphophages ΦVPE25 and ΦVFW. The protein, named PIP_EF_, due to homology to its lactococcal counterpart [114], was found to contain a central, hypervariable region of 160 aa which directly correlates to host range. Investigations into phage resistant isolates which harboured mutations within PIP_EF_ determined that it facilitates DNA injection, while it is not involved in adsorption to the host [119]. Thus overall, successful infection by ΦVPE25 is a two-step process of initial EPA-mediated attachment followed by PIP_EF_-activated DNA injection [115], [119]. Similarly, ΦNPV1 requires both PIP_EF_ and a functional *epa*R for successful infection of *E. faecalis* OG1RF [120]. Of note, it has recently been suggested that additional and, as yet unidentified DNA injection triggers exist for *E. faecalis* phages [117], [121]. A summary of EPA-associated proteins shown to be complicit in phage infection is shown in Table 2.

**Table 2:** Summary of EPA associated proteins which have been shown to be critical for phage infection.

Protein	Function	Location	Phage
EpaA	Initiation of rhamnan biosynthesis	Conserved	NPV1
EpaB	GT- rhamnan elongation	Conserved	NPV1
EpaR	UDP Glc phosphotransferase	Conserved	NPV1, phi4, phi51, phi47, phi19
EpaX	GT- GalNAc decoration of TA chain	Variable	Idefix, phi19, phi4
EpaY	Membrane protein – polymerisation TA blocks	Variable	Phi4
EpaM	ABC transporter – translocation of rhamnan	Conserved	NPV1
EpaN	GT – rhamnose elongation	Conserved	NPV1
EpaE	rmlA – Glc-1-P thymidylyltransferase	Conserved	NPV1
EpaOX	GT- GalNAc decoration of TA chain	Variable	VPE25
EpaOX2	GT	Variable	VPE25
EpaOY	Membrane protein – polymerisation TA blocks	Variable	VPE25
EpaS	GT- GalNAc decoration of TA chain	Variable	Phi4, phi47, phi19
EpaW	Epimerase – conversion to UDP-Rha	Variable	Phi17
EpaAC	Epimerase – UDP-GlcNAc to UDP-GalNac	Variable	Phi4, phi19, phi47

## 2 Summary and outlook

Recent studies have provided significant insights into the biosynthesis of Rha cwps for key ovococcal species. The identification of a direct relationship between gene content and architecture, shared gene function and Rha cwps structure, provides an *in silico* template for predicting key structural features for strains or species where biochemical data is lacking. We have highlighted significant commonalities for both gene function and biosynthesis of ovococcal Rha cwps. It has recently been proposed that the nomenclature of the *S. mutans* serotype C Rgp, and its associated genes, be changed to SCC (sero-specific carbohydrate C) and *scc*, respectively, to better reflect their function [29]. As common intra-species functions have been identified for Rha cwps biosynthetic genes, a future harmonisation of the nomenclature across ovococcal species may be required. Furthermore, the utilisation of shared Rha cwps biosynthesis machinery across both pathogenic and non-pathogenic ovococcal species including that of the rhamnan core, transporter systems, GTs, and membrane proteins reflects a genetic marker which can be used to identify similar loci in bacterial species which have been shown to produce Rha cwps. Model systems for Rha cwps are now well established for the non-pathogenic *S. mutans* and *L. lactis* and, owing to common Rha cwps biosynthetic steps these model systems may now serve as a roadmap to understanding the basis of Rha cwps assembly in as yet uncharacterised pathogenic ovococci. As genomic and biochemical information relating to Rha *cwps*-associated biosynthesis gene clusters is expected to expand, inter-species comparative analysis will further our understanding of ovococcal Rha cwps structure, diversity, biosynthesis, and functionalities. Furthermore, the evolution and genetic diversity of Rha *cwps* loci is heavily influenced by external pressures, in particular, phage predation. Longitudinal and comparative mapping of phage induced mutations in Rha *cwps*-associated genes may serve as a tool for elucidating critical, or indeed, shared elements of biosynthesis in ovococcal species, which mediate phage sensitivity. In addition, the rapid response of phage, at times by single point mutation, to overcome and adapt to Rha cwps modifications allows for the identification and/or characterisation of phage-encoded host recognition machinery which specifically recognise Rha cwps on the host cell surface.

## CRediT authorship contribution statement

**Katherine Lavelle:** Writing – original draft. **Douwe van Sinderen:** Writing – review & editing. **Jennifer Mahony:**Writing – review & editing.

## Declaration of Competing Interest

The authors declare that they have no known competing financial interests or personal relationships that could have appeared to influence the work reported in this paper.
